# A Hypercalcemic Enigma: A Rare Case of Bone Marrow Sarcoidosis

**DOI:** 10.7759/cureus.40534

**Published:** 2023-06-16

**Authors:** Biplov Adhikari, Beisi Ji, Syed Hamza Bin Waqar, Sagun Khatri

**Affiliations:** 1 Internal Medicine, MedStar Union Memorial Hospital, Baltimore, USA; 2 Internal Medicine, State University of New York Downstate Health Sciences University, New York City, USA; 3 Internal Medicine, B.P. Koirala Institute of Health Sciences, Dharan, NPL

**Keywords:** diagnostic dilemma, non-caseating granuloma, hyperthyroidism induced hypercalcemia, multiple myeloma, osseous sarcoid

## Abstract

Sarcoidosis is a multisystem inflammatory disease involving granuloma formation. The exact etiology of the disease remains unknown. While the lungs are the most commonly involved organs in sarcoidosis, bone marrow-only involvement is quite rare. As bone marrow-only involvement can have a presentation that closely resembles multiple myeloma, a significant diagnostic dilemma can occur as the treatment for sarcoidosis is different compared to multiple myeloma. We present a case of a 62-year-old female who presented with worsening lower extremity weakness and fatigue. She was found to have new-onset hypercalcemia, normocytic anemia, and lytic bony lesions. Extensive laboratory workup for multiple myeloma was negative with bone marrow biopsy showing non-caseating granulomas, thus diagnosing the patient with sarcoidosis involving the bone marrow.

## Introduction

Sarcoidosis, coined by Boeck in 1899 after observing epithelioid and giant cells on a skin biopsy, is a multisystem inflammatory disease involving non-caseating granuloma formation [1]. It afflicts patients across the world with varying prevalence due in part to differences in genetics, human leukocyte antigen (HLA) allelic variation, and environmental conditions [2].

This multisystemic granulomatous disease most commonly, in up to 90% of the cases, involves the lungs. Other commonly involved systems include lymph nodes, skin, eyes, and the liver [3]. However, it is quite rare for sarcoidosis to involve the bone marrow with studies showing prevalence ranging from 1% to 13% [4]. As bone involvement can manifest with signs and symptoms that overlap with other hematological diseases, the diagnosis of bone marrow sarcoid can pose a significant diagnostic dilemma. We present a case of bone marrow sarcoid presenting with hypercalcemia which was initially mistaken for multiple myeloma.

## Case presentation

A 62-year-old African American female with a pertinent past medical history of diabetes and hypertension presented to the emergency department with bilateral leg weakness for a week. She did not have a family history of malignancies. The progressive weakness was associated with tingling and numbness. Her history was not concerning fecal or urinary incontinence or saddle anesthesia. She also reported unintentional weight loss of about 10 kg and ongoing fatigue for one month. Vitals at the initial encounter were within normal limits. Physical examination showed mild weakness in bilateral lower extremities.

Initial laboratory workup revealed hypercalcemia (Table 1). Her calcium level two months prior to admission was normal. She also had a new-onset acute kidney injury and a slight normocytic anemia.

**Table 1:** Pertinent laboratory results at presentation and two months prior to presentation. PTH: parathyroid hormone; MCV: mean corpuscular volume.

Test	At presentation	Two months prior to presentation	Reference values
Hemoglobin (g/dL)	11.3	13.5	12.0-16.0
MCV (fL)	88.5	97	78.0-95.0
Calcium (mg/dL)	13.4	9.8	8.8-10.2
Phosphorus (mg/dL)	3.7	Not available	2.5-4.5
Creatinine (mg/dL)	2.12	0.73	0.50-0.90
Total protein (g/dL)	7.0	7.9	6.4-8.3
Albumin (g/dL)	3.2	3.6	2.8-5.7
Alkaline phosphatase (U/L)	227	240	25.0-125.0
Parathyroid hormone (pg/mL)	11.0	Not available	15.0-65.0
PTH-related peptide (pmol/L)	<2.0	Not available	<2
Vitamin D, 25-hydroxy (ng/mL)	24.4	Not available	30.0-100.0
Vitamin D 1,25-dihydroxy (pg/mL)	80	Not available	19.9-79.5
Angiotensin-converting enzyme (nmol/L/min)	111	Not available	<40

Her imaging, including whole-body roentgenograms, showed multiple scattered calvarial lesions as well as lytic lesions on the right and left iliac crest and the left greater trochanter. Given her moderate hypercalcemia, new-onset anemia, and lytic lesion on the skeletal survey, multiple myeloma (MM) was considered the most likely diagnosis. She was then admitted for further management of hypercalcemia.

**Figure 1:**
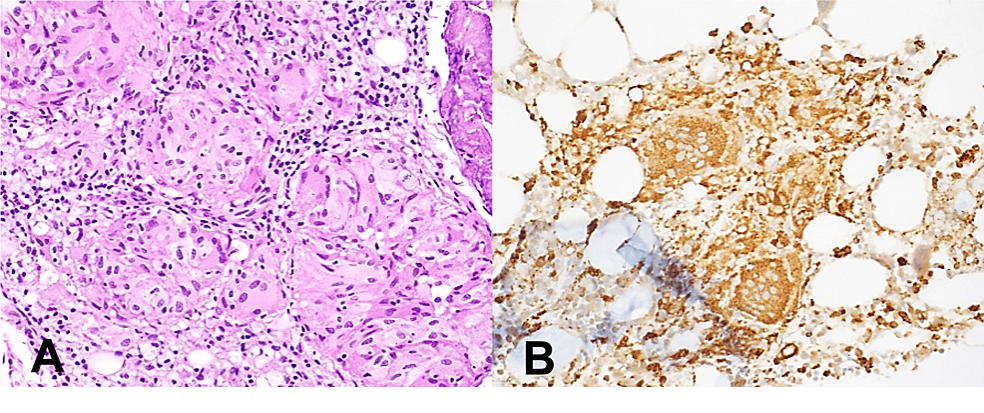
Bone marrow biopsy. A: The bone marrow biopsy shows non-necrotizing granulomas with giant cells (H&E, 400×). B. CD68 immunohistochemistry staining highlighted granulomas. H&E: hematoxylin and eosin.

**Figure 2:**
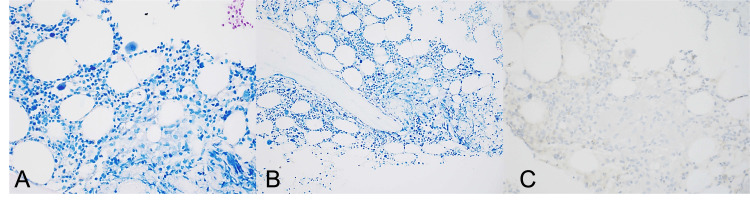
No microorganisms are stained. No microorganisms are stained on AFB (A), Fite (B), and toxoplasmosis special staining (C). AFB: acid-fast bacteria.

She received isotonic fluids, calcitonin, and bisphosphonate with gradual improvement in her calcium levels. Further workup revealed a low parathyroid hormone (PTH) level and slightly elevated 1, 25 dihydroxy vitamin D3 level. Her PTH-related peptide was undetectable. The serum electrophoresis (SPEP) did not show an M-spike; she however did have a polyclonal gammopathy. Her immunofixation was normal, and she did not have abnormal kappa or lambda light chains in her urine and serum. The tests for viral etiologies of anemia including human immunodeficiency virus (HIV), hepatitis B, and hepatitis C were negative. Interferon-gamma release assay (IGRA) was negative. Vitamin B12 and folic acid levels were normal. Given the very high suspicion of MM, further bone scans were done which failed to show any further lytic lesions or pathological fractures. Bone marrow aspiration and biopsy were performed which showed non-necrotizing granulomas, but there was no evidence of plasma cell neoplasm or B cell lymphoma (Figures 1, 2).

At this juncture, given the negative workup for MM, we then pursued an extensive rheumatology workup as she had non-PTH-dependent hypercalcemia and non-caseating granulomas in the bone marrow. The suspicion of sarcoidosis was high at this point. Her angiotensin-converting enzyme was elevated (Table 1). She also had raised inflammatory markers including the erythrocyte sedimentation rate (ESR) (40 mm/hr (reference range: 0 to 20)) and C-reactive protein (CRP) (42.1 mg/dL (reference range: <0.3)). Computed tomography (CT) of the chest, abdomen, and pelvis without contrast, performed to evaluate primary granulomatous focus, showed perihilar reticulation, likely representing sequelae of sarcoidosis. A primary granulomatous focus, however, was never found.

Consequently, she was diagnosed with sarcoidosis of the bone marrow and started on methylprednisolone 120 mg daily, hydroxychloroquine 200 mg twice a day, and ketoconazole 200 mg twice daily with further improvement of calcium. Angiotensin-converting enzyme (ACE) and vitamin D3 levels improved subsequently on the follow-up laboratory results one month later. She was discharged on hydroxychloroquine 200 mg twice daily, a prednisone taper, and azathioprine 100 mg daily. She reported the resolution of her fatigue, weakness, and numbness on her three-month follow-up.

## Discussion

In this case report, we have described a rare presentation of sarcoidosis, involving the bone marrow, manifesting with new-onset non-PTH-dependent hypercalcemia, anemia, and lytic lesion. Our patient’s symptoms had a significant overlap with the symptoms typically associated with MM, thus leading to a diagnostic dilemma. Despite the description of sarcoidosis over a century ago and extensive research, the exact etiology of the disease is yet to be known. Multiple etiologic associations have been hypothesized; however, no single etiologic agent or exact genetic locus has been identified for disease causation [5]. The ACCESS case-control study which involved more than 700 patients and over 30,000 relatives too was unsuccessful at finding the exact etiology of the disease [6]. Occupational exposures including exposure to beryllium, zirconium, and aluminum have been hypothesized as disease triggers, as have infectious agents like mycobacteria and cutibacteria [7].

The immunopathogenesis of the disease involves a complex interplay of immune cells and immune mediators. Granuloma formation occurs consequent to antigen presentation, by antigen-presenting cells, to the cluster of differentiation 4 (CD4)+ T helper lymphocytes, leading to an immune response involving interleukin (IL)-2, interferon-gamma, and other cytokines [8]. Under the microscope, the non-caseating sarcoid granulomas have a tightly packed central area with epithelioid cells, multinucleated giant cells, and macrophages which are surrounded by lymphocytes (both CD4 and CD8), monocytes, and B lymphocytes [9]. In the United States of America (USA), the incidence of sarcoidosis is three times higher in African Americans compared to white Americans. The highest incidence is observed in African American females. It is also associated with worse mortality and morbidity in African Americans [2,10].

Although it can involve all organ systems, lung involvement is present in 90% of cases of sarcoidosis with a predilection for upper lobes. There is often hilar and mediastinal lymphadenopathy [11]. Hypercalcemia is the most common electrolyte abnormality seen in sarcoidosis; it occurs in 10%-20% of cases. Hypercalciuria may be seen in up to 50% of the cases. Hypercalcemia is associated with a more severe disease presentation and is seen particularly among patients with the HLA-DRB1*04 allele [12]. Hypercalcemia is in the setting of increased production of calcitriol by activated immune cells (mainly macrophages) in the granulomas [13]. The presence of hypercalcemia and renal failure without pulmonary finding can present a diagnostic dilemma. In the case of our patient, the diagnosis was further confounded by the presence of anemia, leading us to consider, strongly, the possibility of multiple myeloma in our patient.

Sarcoidosis and multiple myeloma, although very different systemic diseases, can both lead to hypercalcemia. Sarcoidosis does not have a monoclonal gammopathy on electrophoresis. It is important to differentiate the two as hypercalcemia in sarcoidosis can be treated with glucocorticoids. Other agents like ketoconazole, chloroquine, and hydroxychloroquine can also be used to treat hypercalcemia in sarcoidosis [14]. Steroids lower calcium through the suppression of calcitriol production in granulomas, while hydroxychloroquine and ketoconazole inhibit 1-α-hydroxylase [15]. Our patient was treated with hydroxychloroquine and ketoconazole.

As is often in medicine, abnormal presentations of diseases can lead us to diagnostic dilemmas. There are also case reports of sarcoidosis concurrently with or followed by multiple myeloma [16]. Indeed, sarcoidosis is associated with a higher risk of lymphoproliferative disorders [17]. Rarely, sarcoidosis can also present as a paraneoplastic syndrome secondary to multiple myeloma [18]. In the case of our patient, extensive investigation including bone marrow biopsy was negative for multiple myeloma.

## Conclusions

Sarcoidosis can rarely involve the bone marrow and present with lytic lesions, anemia, and hypercalcemia features common to multiple myeloma. Sarcoidosis should be considered as one of the differentials if the laboratory workup for multiple myeloma is negative as the treatment of either entity is different.
